# First steps towards distinguishing Mediterranean vegetation root marks on bones: An experimental approach

**DOI:** 10.1371/journal.pone.0351568

**Published:** 2026-06-18

**Authors:** Alba Macho-Callejo, Dores Marin-Monfort, Aida Gutiérrez, Sara García-Morato, Yolanda Fernández-Jalvo

**Affiliations:** 1 Departamento de Biodiversidad, Ecología y Evolución, Universidad Complutense de Madrid, Madrid, Spain; 2 Departamento de Paleobiología, Museo Nacional de Ciencias Naturales (MNCN-CSIC), Madrid, Spain; 3 Departamento de Medicina, Facultad de Medicina, Salud y Deporte, Universidad Europea de Madrid, Madrid, Spain; 4 Red Iberoamericana de Investigadores Forenses (RIIF), CYTED, Programa Iberoamericano de Ciencia y Tecnología para el Desarrollo (2021), Madrid, Spain; 5 INGEOSUR, Departamento de Geología, Universidad Nacional del Sur (UNS)-CONICET, Buenos Aires, Argentina; 6 Departament de Botànica i Geologia, Universitat de València, Valencia, Spain; 7 Independent Anthropologist, Present Address: Genetika, Antropologia Fisikoa eta Animalien Fisiologia Saila, University of the Basque Country, Santsoena, Spain; 8 Instituto de Historia, Dpto de Arqueología y Procesos Sociales, Grupo de Investigación en Paleoeconomía y Subsistencia de las Sociedades Preindustriales, Madrid, Spain; University of Haifa, Zinman Institute of Archaeology, ISRAEL

## Abstract

Root-bone interactions are common in buried skeletal remains, yet their diagnostic value remains largely unexplored because few controlled studies have linked root marks to specific plant types. Consequently, the potential of these marks to provide information about burial environments in archaeological, paleontological, and forensic contexts has been largely overlooked. Here, we present a long-term experimental study documenting root-induced bone modifications under natural field conditions in central Spain. Deer ribs were buried at various depths and for different lengths of time among three widespread Mediterranean trees and shrubs: holm oak (*Quercus ilex*), olive (*Olea europaea*), and grapevine (*Vitis vinifera*). Using optical and scanning electron microscopy, we identified distinct patterns of root engraving on cortical bone surfaces that varied by plant type. Holm oak roots produced sinuous, dendritic grooves; olive roots generated shallow, rectilinear markings; and grapevine roots formed linear-to-circular engravings, which were often associated with localized cracking. Mark intensity increased with burial depth and duration. These findings underscore the diagnostic value of root marks in identifying plant-specific signatures and offer a novel approach to recognizing plant activity in burial environments. This information improves taphonomic interpretations in various fields, including fossil reworking processes and forensic secondary burials.

## Introduction

Vegetation provides essential information about the environment in which skeletal remains are deposited and offers invaluable insights into past environments and climates. Reconstructions of past environments and paleoclimatic changes are often inferred from direct botanical remains, such as charcoal, seeds, wood, pollen, and phytoliths from plants, fungi and algae [1–4]. Archaeobotanical studies shed further light on the interactions between plants and humans. Phytoliths preserved in dental plaque or tartar record interactions between humans or other animals, and plants. They are incorporated through diet and plant processing. They provide evidence of consumed plants and some non-dietary activities, such as old fuel management (e.g., charcoal) since phytoliths make it possible to distinguish between different parts of plants, allowing us to determine whether firewood or other materials, such as grass, were used to fuel fires, seed exploitation, and the spatial organization of human populations [5–9]. In addition, stable carbon isotope (δ¹³C) analyses, obtained from the tissues of herbivores, carnivores, rodents and hominins, provide information on the proportions of C₃ versus C₄ plants ingested and/or present in the ecosystem that may reinforce plant type identifications. This research provides insights into subsistence strategies and available vegetation in past ecosystems, offering basic data on the diet, behavior, and nearby habitats of early hominins [10–14].

In forensic contexts, on the other hand, botanical and palynological studies contribute to locating clandestine graves and identifying secondary burials, determining some causes of death (e.g., accidental death, murder or suicide) and estimating the *post-mortem* interval or PMI [15–24].

Taphonomy, originally developed in paleontology, has become a central tool in archaeology and, more recently, in forensic sciences [15,16,25–37]. This discipline plays a significant role in interpreting depositional environments and distinguishing between natural and anthropogenic modifications [20,22,23,38–42]. Among the various post-depositional agents, vegetation is particularly relevant because roots frequently leave visible marks on bones in paleontological, archaeological, and forensic contexts. Despite their frequent mention in taphonomic descriptions, root marks are rarely characterized in detail [23,26,43–47]. Consequently, the specific types of plants responsible for these modifications remain poorly understood. Recent experimental and monitoring studies have begun to address this gap [48,49] aiming to compare modern reference samples (experimental-neotaphonomic) with fossil and historical specimens (real cases). However, a more systematic characterization of root-induced bone modifications is needed to accurately interpret the depositional environments and climates in all these contexts.

Plant roots interact with buried bones to acquire essential nutrients for growth, leaving distinct marks on the bone surfaces. Through the exudation of acids, roots increase the availability of phosphates from bones, which they then metabolize. The root marks differ depending on the type of plant (herbaceous, shrub, or tree), soil, climate, and root morphology [50–53]. Several authors have attempted to develop classifications and characterizations of these marks in the fossil record [33,46,54–56] but detailed long-term experimental studies involving known vegetation types remain scarce. Our recent research with herbaceous taxa (Bermuda grasses, common grasses, and cattails) has shown a direct relationship between the morphology of root engravings and the specific plant species responsible, thereby providing insights into the depositional environment [48].

Based on this previous work, the present study expands this experimental framework to include Mediterranean trees and shrubs, in order to evaluate whether woody taxa produce root marking patterns that differ from those produced by herbaceous taxa. Specifically, we aim to characterize root marks on the cortical surface of bones buried in soil surrounding different and known plants and considering other variables such as burial depth and burial time. Although identification at the genus or species level is not yet possible, this experimental study is a necessary step toward refining the interpretive potential of root marks caused by trees and shrubs and how these differ from marks caused by herbaceous plants. The study may generate a database of root marks that will allow us to easily determine whether skeletal remains were buried near trees, shrubs and/or herbaceous plants. This underscores the value of root marks in paleontological, archaeological, and forensic contexts.

### The relevance of identifying vegetation traces

Vegetation is a key component of the environment, and identifying the traces left by different plant types on bone is therefore essential for reconstructing past environments and interpreting depositional contexts. Root activity varies between functional plant groups: herbaceous plants secrete larger amounts of organic acids compared to trees and shrubs [50–53,57], which enhances their capacity to dissolve bone mineral and accelerate surface etching. This greater decomposition potential may explain the more aggressive patterns observed in our previous study with herbaceous vegetation, in contrast to the comparatively shallow or localized modifications produced by arboreal roots. Such differences highlight the importance of considering plant physiology and root exudation dynamics when interpreting root-related marks. In fact, Galligani [56] documented fine, sinuous root marks linked to herbaceous, low-growing vegetation, in agreement with our earlier findings [48].

In numerous taphonomic studies in paleoecology and archaeology, root marks are reported among post-depositional modifications on bones, yet their precise origin is seldom examined in depth. Nevertheless, their presence has informed multiple interpretative frameworks but are rarely subjected to detailed morphological or causal analysis. Root marks on skeletal remains may indicate vegetation growth in stratigraphic levels or in cavities where plant development would otherwise be unexpected [58–61]. They may also provide information on the succession of taphonomic events, suggesting possible processes of transport or reworking of skeletal remains [58–61].

The identification of root marks has made it possible to determine whether a cave was at some point exposed to the open air or whether an underground gallery was connected to the outside environment, providing key information on the site formation and evolution of the fossil sites [62]. Furthermore, their presence or absence can be linked to patterns of human activity, helping to interpret the dynamics of occupation and abandonment of particular sites [63]. The association of these marks with wet periods has been documented in many studies, which reinforces their usefulness as an indirect indicator of past climatic conditions [64,65].

In forensic anthropology, the distinction between different taphonomic modifications (such as cut or tooth marks, and insect or vegetation modifications) is essential to distinguishing between peri- and *post-mortem* injuries [66]. In this context, experimental evidence indicates plant-type-dependent differences in root marks, which may be relevant for understanding potential movement of bones/skeletons found in secondary mortuary burials [48]. Although some authors indicate that cadaveric remains from forensic contexts do not show root marks because of the short time elapsed [67], experimental and prospective studies have documented their occurrence, even within relatively short burial intervals [48,49,58,68–72].

It is important to take also into consideration the symbiotic relationships between plants and fungi (*mycorrhizae*) or bacteria (*rhizobia*) for nitrogen fixation and nutrient acquisition improve plant growth in adverse environmental conditions. In fact, olive trees are associated with several types of fungi for greater efficiency in arid climates [73,74]. In relation to root activity associated with fungi, dark marks (brown patches) have been recorded on the cortical surface, similar to those observed in samples buried near grapevines and holm oaks. However, no associated hyphae were observed in these samples [71,75].

## Materials and methods

### La Higueruela experimental field site

La Higueruela (https://mncn.csic.es/es/investigaci%C3%B3n/servicios-cientifico-tecnicos/finca-experimental-la-higueruela) is an experimental field station of the Spanish Research Council (CSIC) and it is located ~80 km south of Madrid (40°3’21.80″ N/ 4°25’31.13″ W), at an elevation of 486 m asl. This site is an example of a semi-arid Mediterranean environment, characterized by low mean annual precipitation (below 650 mm, particularly less than 60 mm/month during summer), a pronounced dry season, and mean annual temperatures ranging between 14 and 15.5°C.

Since 2012, a long-term taphonomic experimental project has been conducted at La Higueruela. Bones from large mammals (deer ribs), small mammals (rodent carcasses), and pellets (from barn owls) were buried under various conditions to investigate bone modifications after deposition: interaction with plant roots, pH conditions and soil horizons. The samples buried in the taphonomic study of interaction with plant roots were distributed across different fields with different plant species: [1] the taphonomic station; [2] the olive grove; [3] the holm oak forest; and [4] the vineyard. The distribution of the samples is summarized in Table 1.

**Table 1:** Distribution of buried samples for the taphonomic study of interaction with plant roots, including sample type (deer ribs, rodent carcasses, and pellets) and the number of samples analyzed in this study.

Experimental area	Total samples	Deer ribs	Rodent carcasses	Pellets	Samples of this work
**1. Taphonomic station**	39	13	13	13	0
**2. Olive grove**	36	12	12	12	3
**3. Holm oak grove**	18	6	6	6	2
**4. Vineyard**	9	3	3	3	2

**Fig 1:**
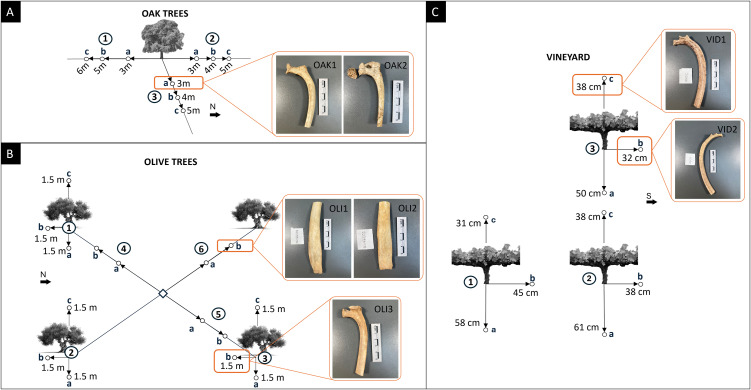
Distribution of burials in La Higueruela experimental areas. **A:** holm oak trees, **B:** olive trees and **C:** vineyard. Orange squares indicate the rib samples analyzed in this study at different distances from the vegetation. The numbered circles and labels (a, b, and c) indicate the areas where the samples were buried.

One part of this project focuses on the effect of Mediterranean woody vegetation on 21 experimental burials of red deer ribs (*Cervus elaphus* Linnaeus, 1758*)*, placed beneath holm oaks, olive trees and grapevines (Fig 1). This paper focuses on a subset of this experiment, specifically seven ribs from an adult female deer buried in contact with these three plant types. The remaining ribs remain buried for future recovery.

### Experimental design

Ribs were chosen for this project because they are one of the most abundant skeletal elements and help minimize individual variability. Furthermore, deer bones have been used in other forensic studies [41], which would allow us to conduct comparative studies in archaeological and forensic contexts.

All samples were collected from naturally skeletonized deer carcasses. Prior to burial, ribs were cut into two pieces using a diamond saw. The samples were photographed and examined under a binocular light microscope (Leica M205A) to rule out pre-existing bone modifications on the cortical surface, such as osteological features, pathologies, microbial corrosion, weathering, animal chewing or sample preparation damage. This step ensured that all observed modifications were post-burial alterations.

The burial of the seven ribs studied here was carried out in 2012 under different types of trees and shrubs at different depths and distances (Table 2). An additional set of 14 ribs were buried under the same conditions and left in place for a longer-term exposure, to be recovered after extended burial intervals (Table 2; Fig 1). Plants were selected based on their root system structure and growth dynamics, including tall and low-growing evergreen trees, and rapid root growing shrubs. While holm oaks and olive trees are native Mediterranean species, grapevines originate from temperate climates but are well adapted to high temperatures. Holm oaks (*Quercus ilex*) were chosen as a tall tree with a robust taproot system with deep primary roots from which lateral roots develop and located on clay soil, which are known for their high water and nutrient retention but poor drainage, causing the soil to compact when wet and deep cracks when dry. Olive trees (*Olea europaea*) were chosen as a low-growing tree species, characterized by an extensive shallow root system consisting of a single primary root with shallow secondary roots and they were located in sandy soil, with coarse-grained particles that allow for good water drainage. Grapevines (*Vitis vinifera*) were chosen as a shrub species with tap roots and both underground and adventitious secondary roots [76]. These plants were located in mixed sediment soil (a combination of clay, sand, and silt), which provides good aeration as well as good water and nutrient retention. Friction against sediment, particularly sands, could scratch the bone surface, but such marks are linear and of mechanical nature, different from grooves produced by roots [66].

**Table 2:** Sample codes and contextual information for each plant species and burial condition. Plant distance refers to the distance between the buried bone sample and the plant trunk.

Sample code:	Plant	Depth	Distance to plant’s trunk	Soil pH	Root system	Time interval
**OAK1**	Holm oak	25 cm	3 m	5	Taproot and lateral roots	1 year
**OAK2**	Holm oak	50 cm	3 m	5	Taproot and lateral roots	1 year
**OLI1**	Olive tree	25 cm	1.5 m	7	Extensive shallow	1 year
**OLI2**	Olive tree	50 cm	1.5 m	7	Extensive shallow	1 year
**OLI3**	Olive tree	50 cm	1.5 m	7	Extensive shallow	10 years
**VID1**	Grapevine	40 cm	38 cm	6	Subterranean & adventitious	1 year
**VID2**	Grapevine	40 cm	32 cm	6	Subterranean & adventitious	3 years

Before burial, locations were selected based on plant types. Cylindrical boreholes were drilled to specific depths, and samples were buried at 25 and 50 cm, or 40 cm (Table 2). All samples were placed near their respective plant species but at varying distances from them depending on the size of the plant (Table 2, Fig 1). In the case of the holm oaks, six ribs were buried at 3 m, 4 m and 6 m from the tree trunk (Fig 1A), two of which are analyzed here. Twelve samples were buried near olive trees at 1.5 m distance (Fig 1B), three of them are studied here. Three samples were buried approximately 30 cm from grapevines (Fig 1C), two of which are included in this study. Therefore, the seven ribs analyzed in this paper remained buried from periods ranging from 1 to 10 years (Table 2). When the samples were buried, the goal was to recover them after one year, 10 years, and 15 years. However, due to practical constraints during the experiment, such as plant mortality (e.g., vines) and the loss or displacement of some samples (e.g., due to root growth or animal activity), it was not possible to recover all specimens at the planned intervals.

### Collection, preparation and analysis

Samples were excavated at intervals of one, three, and ten years after burial to examine whether the time of burial was related to the intensity of the marks. Plant roots and surrounding sediments were also collected for comparative analysis. Soil pH is monitored at the Field Station according to the edaphic layer and location within the Higueruela Station. We also used pH strips measured both near the samples and in control areas to assess variations in acidity and to determine its effect on the bones compared to that on the roots. The climate data (temperature and precipitation) for the burial period (2012–2022) are shown in Table 3. These values are characteristic of a semiarid Mediterranean climate, characterized by high temperatures in the summer (an average of 25.6°C) and low temperatures in the winter (average of 7.4°C), as well as low precipitation (average of annual accumulated rain 380 mm and 35.19 mm monthly). Some years have recorded even less rainfalls. Of course, these variations may have influenced root activity and soil moisture, thereby affecting the development and intensity of root marks, although variations observed during the time span of the experiment may not be extreme within this semiarid environmental context (average of annual accumulated rain 300–700 mm).

**Table 3:** Average values of climate variables (precipitation and temperature) by year Temperature in degrees Celsius (°C) and precipitation in millimeters (mm).

Year	Temperature (annual average; ºC)	Precipitation (annual accumulated mm)
**2012**	15.7	312.5
**2013**	15.7	472.0
**2014**	15.3	464.3
**2015**	13.3	403.4
**2016**	16.2	511.5
**2017**	17.7	466.6
**2018**	18.2	561.3
**2019**	16.0	317.7
**2020**	16.1	369.6
**2021**	15.7	444.4
**2022**	17.4	464.8
**Mean**	16	435.3

All specimens were transported in perforated plastic or paper bags to minimize moisture accumulation and prevent microbial proliferation. Bone samples were cleaned at the Laboratory of Environmental Analysis and Taphonomy (LeaT) of the MNCN-CSIC using distilled water in an ultrasonic bath for a maximum of three minutes, in order to avoid potential surface abrasion and rounding of bones caused by prolonged exposure to ultrasonic waves [48]. The cortical surface of each bone was analyzed under a binocular optical microscope (BOM) with automated Z-focus (LEICA M205A), equipped with a high-resolution digital camera (LEICA DFC 450), which provided detailed photographs of observed features. Selected areas of interest were further analyzed using a scanning electron microscope (SEM; FEI INSPECT) housed at the Non-Invasive Techniques Laboratory of the MNCN-CSIC (LTND). A low-vacuum SEM was employed, allowing imaging without gold or carbon sputtering. Images were captured at multiple magnifications in both backscattered and secondary electron detection modes.

**Fig 2:**
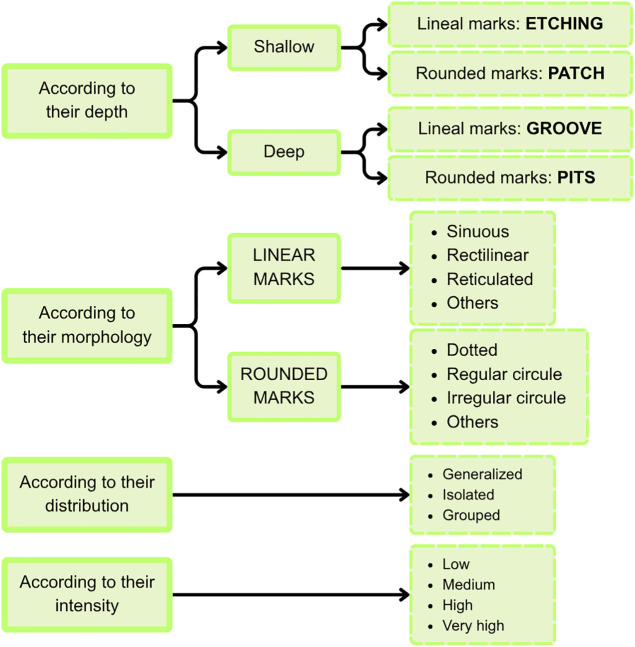
Classification of root marks for linear and rounded marks according to depth, morphology, distribution and intensity over the cortical surface of the bones.

The morphological characterization of root marks was adapted from Macho-Callejo et al. [48] and is summarized in Fig 2. Root marks were grouped into two main types: linear and rounded marks. They were further categorized based on three criteria: depth, morphology, and spatial distribution of the marks. According to depth, marks were classified as shallow (etching and patches) or deep (grooves and pits). Based on morphology, linear marks were distinguished as sinuous, rectilinear, or reticulated, while rounded marks were described as dotted, regular circles, or irregular circles. Spatial distribution was recorded as generalized (covering most or all the cortical surface), isolated (few and widely spaced marks), or grouped (several marks concentrated in a specific area). Finally, the percentage of bone surface affected by root marks was recorded to assess the intensity (low: 0–25%, medium: 26–50%, high: 51–75%, and very high: 76–100%), adapted from Martinez et al. [77].

## Results

Root-marking morphology patterns were distinguished as linear or rounded marks, with clear differences among the selected plant species. The results are summarized in Table 4 and subsequently described in the following subsections for each plant. The analysis conducted in this study is primarily qualitative, which is appropriate given the limited sample size and the exploratory nature of this work. While quantitative measurements (e.g., groove dimensions or mark density) could potentially enhance comparisons between plant contexts, the current sample size does not support statistically robust analyses. The qualitative approach adopted here allows for a consistent characterization of root-mark morphologies across samples. Future studies incorporating larger sample sizes and quantitative measurements will help to refine these observations and strengthen comparative analyses.

**Table 4:** Characterization of root marks observed in deer ribs from La Higueruela according to morphology, distribution and depth. The intensity was assessed based on marks visible to the naked eye. Abbreviations: SEM: Scanning Electron Microscope; BOM: Binocular optical microscope.

Sample code	OAK1	OAK2	OLI1	OLI2	OLI3	VID1	VID2
**Plant**	Holm oak	Holm oak	Olive tree	Olive tree	Olive tree	Grapevine	Grapevine
**Depth (cm)**	25	50	25	50	50	40	40
**Time interval (years)**	1	1	1	1	10	1	3
**LINEAR MARKS**							
**Morphology**	Sinuous	Sinuous	–	Rectilinear	Rectilinear	Rectilinear/other	Rectilinear/other
**Deep**	Grooves	Grooves	–	Etching	Etching	Etching/ Groove	Etching/ Groove
**Distribution**	Isolated	Isolated	–	Generalized	Generalized	Generalized	Generalized
**Intensity**	Medium	High	–	Low	Medium	Medium	High
**Visible under**	SEM	SEM	–	Naked eye	Naked eye	BOM/SEM	BOM/SEM
**ROUNDED MARKS**							
**Morphology**	Dotted	Dotted	–	–	–	Irregular	Irregular
**Deep**	Pits	Pits	–	–	–	Patch	Patch
**Distribution**	Generalized	Generalized	–	–	–	Generalized	Generalized
**Intensity**	High	Very high	–	–	–	Medium	High
**Visible at**	Naked eye	Naked eye	–	–	–	Naked eye	Naked eye

### Holm oaks

**Fig 3:**
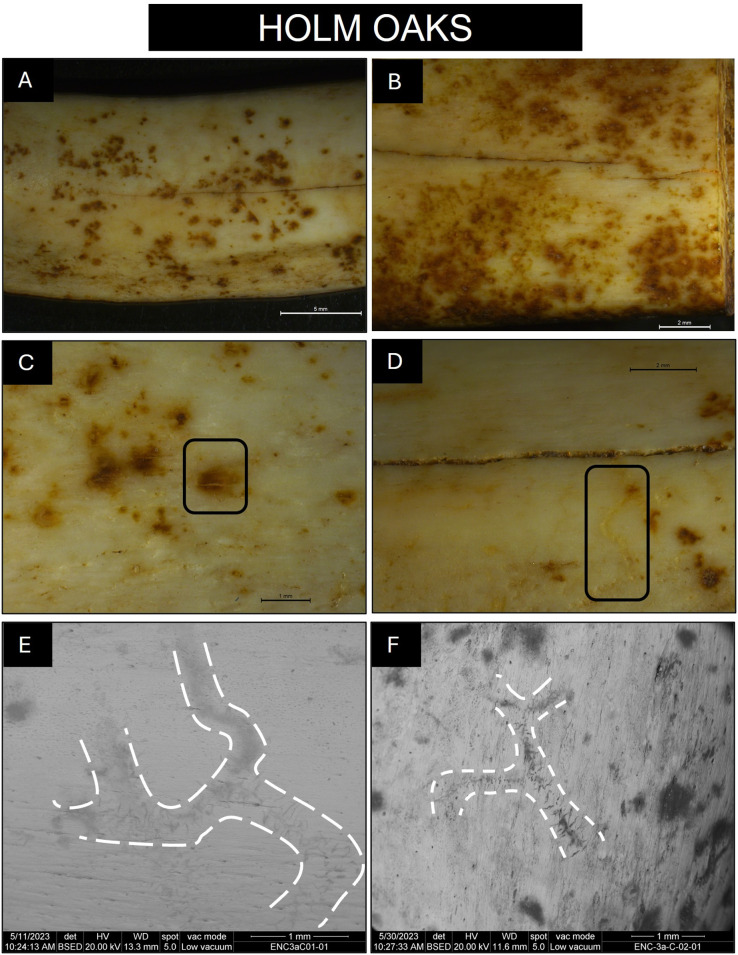
Images of root marks in the cortical surface buried near holm oak roots for 1 year (BOM A to D, and SEM images: E and F). **A, B**: Brown patches on the ribs buried at 25 cm and 50 cm, respectively (OAK1 and OAK2). **C**: Cracking inside the patches (OAK1). **D**: Straight, isolated, sinuous grooves (OAK1). **E, F**: Isolated grooves with irregular edges of buried sample, (OAK1 and OAK2, respectively).

The two samples buried under holm oaks, OAK1 (25 cm deep) and OAK2 (50 cm deep) were recovered after one year. These ribs exhibited similar surface marks with a greater number observed on the deeper sample (OAK2 than on OAK1; Fig 3A and 3B). Microscopic analysis using a BOM revealed both rounded and linear marks on OAK1 and OAK2. The rounded marks appeared as closely spaced dots, some of them penetrating more deeply into the cortical surface than others. These features were more abundant on the deepest buried rib (OAK2), where they appeared as a continuous brown patch, with an irregular outline. Despite the acidic soil pH (pH = 5), cracking was restricted to areas associated with root grooves rather than being randomly distributed across the entire bone surface (Fig 3C). Regarding linear marks, isolated fine short grooves were observed in various areas of both ribs (Fig 3C and 3D; black square). SEM analysis revealed that these linear marks display a sinuous morphology, characterized by multi-lateral micro grooves and irregular edges (Fig 3E and 3F). These grooves were associated with the brown patches (color changes) observed under the optical microscope (BOM).

### Olive trees

**Fig 4:**
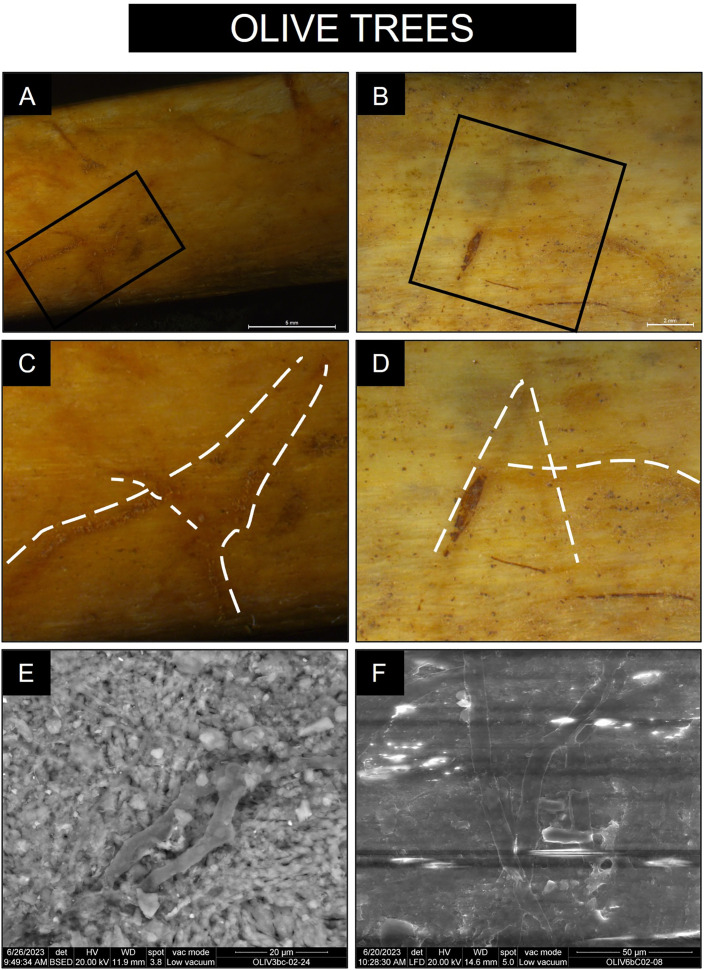
Images of root marks in the cortical surface buried near olive tree roots for one (OLI1, OLI2) and 10 years (OLI3) (BOM images A to D, and SEM images E and F). **A, B**: Linear marks on surface bone of samples OLI2 and OLI3 respectively. **C, D**: Higher-magnification views of the same linear marks, illustrating the “A-shaped” pattern: **E, F**: Fungal hyphae on the cortical surface of sample OLI2.

Specimens OLI1 (25 cm deep) and OLI2 (50 cm deep) were recovered from the olive field after one year of burial, whereas OLI3 (50 cm deep) was exhumed after 10 years. Root-related modifications were observed only in ribs buried at greater depth (50 cm), while the rib buried at 25 cm (OLI1) did not exhibit any root marks. The two marked ribs (OLI2 and OLI3) displayed superficial linear marks characterized under BOM by rectilinear to slightly sinuous grooves, occasionally forming intersecting patterns that produced an “A-shaped” configuration (Fig 4A-4D). These marks were generalized on the cortical surface, although they were more abundant on the rib that remained buried for a longer time (OLI3). Overall, linear marks were shallow and not visible under SEM. Nevertheless, SEM analysis revealed fungal hyphae on the cortical surface of OLI2 (Fig 4E and 4F). These observations suggest that the formation of the marks was influenced more by burial depth than by the duration of burial.

### Grapevines

**Fig 5:**
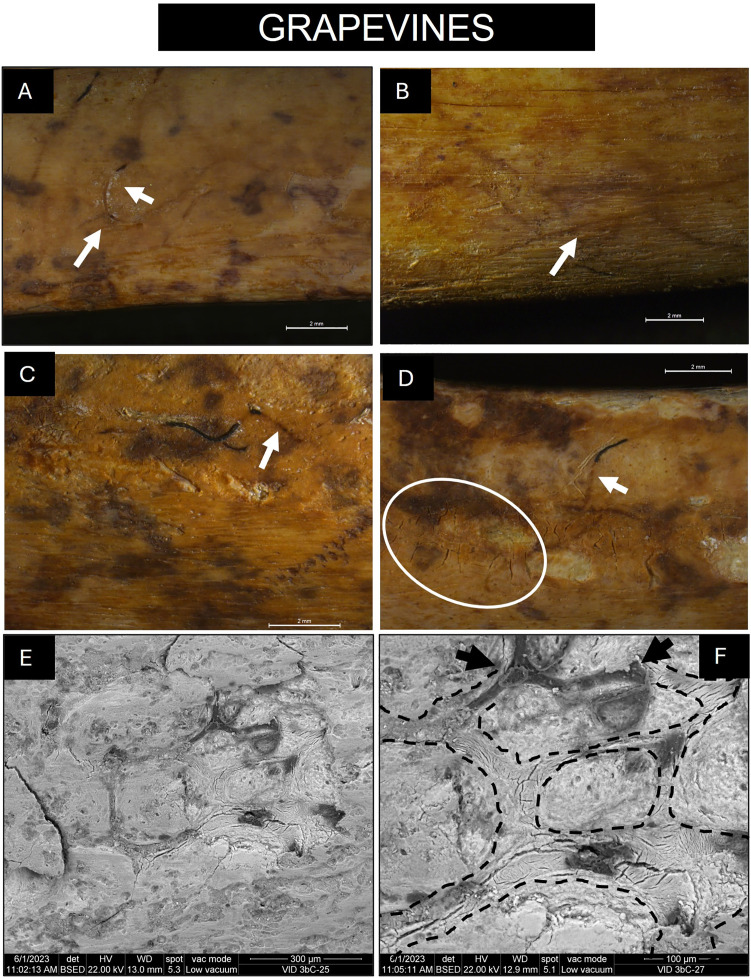
Images of root marks in the cortical surface buried near grapevine roots for one (VID1, A) and three years (VID2, B) (BOM images A to D, and SEM images E and F). **A, B:** Linear root marks on the ribs buried for one year (VID1) and three years (VID2), respectively **C, D**: Linear marks (white arrows), dark brown patch and surface cracks (white circle). **E:** Grooves on rib surface (VID2). **F**: Cracks within the grooves (root marks marked with dotted lines) and remains of the root still attached to the bone (black arrows) in sample VID2.

Two ribs buried in contact with grapevine roots were recovered after one year (VID1; 40 cm deep) and three years (VID2; 40 cm deep) of burial. In both cases, ribs exhibited dark brown patches with irregular morphology, which were widely distributed. These marks were easily visible to the naked eye and were more abundant on the rib that remained buried for a longer time interval (VID2). Vegetation corrosion and surface cracks were also observed (Fig 5C and 5D; white circle). Analysis with a BOM revealed the presence of fine, shallow linear marks (“etchings”) (Fig 5; white arrows). In addition, small, isolated crescent-shaped grooves were observed on both ribs (Fig 5A; white arrow). SEM analysis revealed that several root marks showed the actual root still attached to the bone (Fig 5E and 5F). Cracks were observed inside the root mark, associated with vegetation corrosion (Fig 5F). Although the marks were more abundant on the bone with longer burial, other characteristics remained consistent, suggesting that burial depth may have a greater impact on root-related modifications than the duration of exposure.

## Discussion

The present experimental taphonomic study provides new insights into the burial environment through the analysis and characterization of the marks left by the roots of trees and shrubs. The plant types selected for this work (holm oaks, olive trees and grapevines) are representative of the Iberian Peninsula and typical of Mediterranean vegetation [78,79]. In a previous study [48], we analyzed root marks produced by some herbaceous plants. In the present study, we extend this analysis to include trees and shrubs in order to assess whether root marks on bone remains differ between these plant groups and herbaceous taxa. This experimental approach enables us to make a controlled comparison of root mark morphologies produced by woody versus non-woody vegetation for the first time. The results of this study show that root marks produced by trees and shrubs on buried bones exhibit distinct morphological patterns and also differ from those produced by herbaceous plants.

Roots of holm oaks (*Q. ilex*) generated sinuous grooves and dotted brown pits, were more intense and deeper on the sample buried more deeply. These sinuous marks with irregular edges (Fig 3E and 3F) are similar to those recorded by Thompson (p. 61; Fig 3) [54], who classified them as dendritic marks. In both cases, these marks had a main line from which smaller ones radiated, although, in Thompson’s work [54] they were not associated with any specific vegetation type.

Olive trees (*O. europaea*) roots left rectilinear etchings only on the ribs buried at greater depths (OLI2 and OLI3; 50 cm) and there was more intensity in the sample that remained buried for longer time period (10 years; OLI3). In all cases, they were dispersed along the entire length of the rib (generalized). Indeed, SEM analysis also revealed the presence of fungal hyphae fragments in these samples. Although this observation may be consistent with symbiotic relationships between plant roots and fungi (mycorrhizae) involved in nutrient acquisition and stress tolerance [73,74], a direct causal relationship between fungal activity and bone modification cannot be established based on the present data. Further analyses are required to clarify the role of fungal associations in root-induced bone alterations.

Grapevine (*V. vinifera*) roots left both linear and circular marks. The dark brown patches are similar to those reported by Orlowska [71], who associate them with mycorrhizal activity. However, in the present study, no fungal structures or mineral deposits were detected on the bones surfaces buried under grapevines. Under optical microscopy, fine secondary root traces were observed, and SEM images revealed cracking within the root marks (Fig 5F). Both oak and olive trees develop deep root systems in arid environment [80,81] whereas olive trees in our experimental setting exhibited comparatively shallower lateral roots. Grapevines also possess a taproot root system and may develop adventitious roots [82]; however, no evidence of aerial or adventitious roots involvement was observed in this experiment.

A relevant finding is that even after only one year of burial, both oaks and grapevines left distinctive root marks, highlighting the potential of these taphonomic studies also in forensic contexts, where burial periods are shorter than in paleontological or archaeological settings. Nonetheless, these marks were deeper and denser in samples that remained buried for longer periods (VID2 for three years). On historical, archaeological, and paleontological timescales, root marks can be considered contemporaneous with early post-burial processes. Therefore, the plants that produced these marks reflect the environment at the time of burial or shortly thereafter, providing data that can be compared with dietary reconstructions (e.g., from stable isotopes) or used as direct indicators of local vegetation and climatic conditions. Furthermore, the higher intensity of marks observed in samples buried at 50 cm suggests a relationship between burial depth and the intensity of root-induced taphonomic damage, particularly for olive and oak contexts. In some cases, recently formed marks displayed an isolated distribution.

The relationship between the intensity of marks with depth of burial and time corroborates our previous studies with herbaceous plants [48] and recent descriptions by other authors [49]. However, in the present study, the root marks produced by olive trees were shallow etchings, even after prolonged burial. Color alterations on bone surfaces are commonly interpreted as the result of chemical dissolution processes induced by root contact (red-brownish stains with corroded patches), and grooves may appear as dendritic or linear patterns with a U-shaped cross section, as the root penetrates deeper into the cortical bone [29,46,83]. Corrosion appeared primarily within the grooves, suggesting a spatial restriction of the alteration to root-marked areas and its correspondence with groove morphology. In contrast, soil-related corrosion typically produces more diffuse or less spatially constrained patterns [66]. Finally, these marks can be distinguished in many cases based on their morphological characteristics (e.g., groove cross-section, edge definition, and distribution patterns) from other taphonomic modifications, such as tooth marks or cuts, insect damage or microbial attack [66]. However, in natural contexts, multiple taphonomic agents may act simultaneously or sequentially, and overlapping and partially obscure some features.

**Fig 6:**
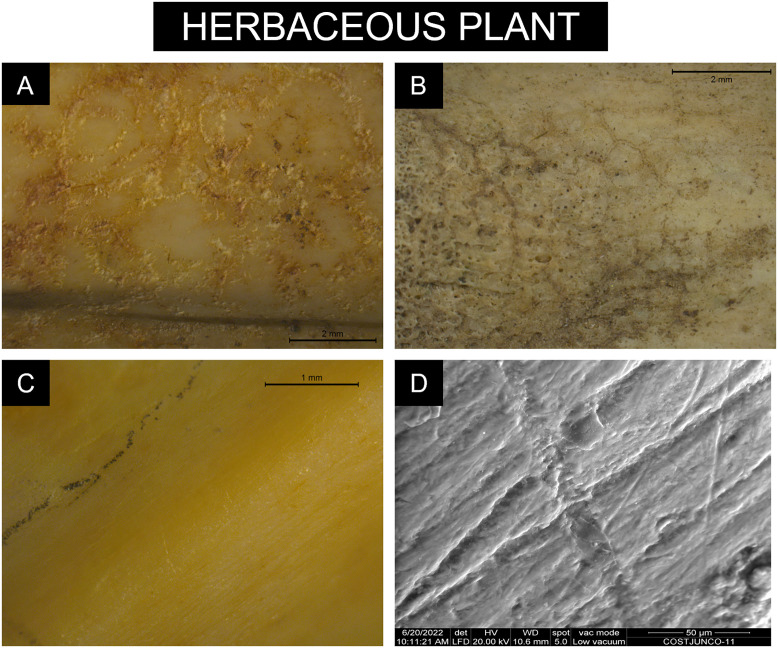
Images of root marks in the cortical surface of deer ribs in contact with herbaceous roots: A: grass (*Dactylis glomerata*) underground adventitious roots. **B:** Bermuda grasses (*Cynodon dactylon*) subaerial adventitious root in rhizome**. C:** Common cattails (*Typha latifolia*) aquatic adventitious roots in fibrous rhizome. **D:** SEM micrograph of the linear and corroded internal mark made by the aquatic cattails (all figures are © Alba Macho-Callejo Ph. Doctoral Thesis).

In this sense, we acknowledge that other plants, such as grasses and weeds, may coexist in natural environments, resulting in an overlap of root systems. However, our previous experimental work with herbaceous plants (https://doi.org/10.1080/08912963.2023.2263865) has shown that these taxa produce distinctive morphological patterns, such as sinuous grooves and characteristic microscopic features (see Fig 6), which differ from the marks produced by tree or shrub roots. Furthermore, in our experiments, specimens are buried at different depths, helping us to determine which marks originate from herbaceous plants and which from the selected trees, since tree roots tend to penetrate deeper. The differences observed in the morphology, depth and spatial distribution of the marks are consistent with those produced by woody taxa and differ from the herbaceous patterns described above. Therefore, while a certain degree of overlap between root systems cannot be entirely ruled out, the main types of modification identified can be distinguished.

Considering the type of plant, we have also examined bones buried with Crassulaceae plants (CAM plants), but no root marks were observed on the bone surfaces, either with the naked eye or under light or electronic microscopy (unpublished personal data). This root-marking absence is likely related to cortical dissolution caused by the exudation of organic acids from roots, which facilitates the release of phosphates for absorption [31,57,84] or to the length of the roots, which in the case of Crassulaceae are short and superficial and, therefore, may not have reached the bone samples or may not be corrosive to the bone surface. Likewise, it has also been proposed that the association of fungi with plant roots may play a key role in bone corrosion, as described by Grayson [75], as well as in the recent findings of Orlowska [71]. Future work, including histotaphonomic analyses, will be necessary to evaluate these aspects.

The results presented here are valuable in paleoecological, archaeological, and forensic contexts. The intensity of root marks can provide information about wet or dry periods in the past, while characterizing the marks and linking them to specific vegetation groups can reveal the type of vegetation cover present at a site, as well as climatic indications. Likewise, in forensic contexts, distinguishing the type of root marks is essential for identifying burial sites or crime scenes, and for understanding the original burial context as well as the potential movement of bodies in secondary burials.

Although this experimental study has certain limitations, particularly the small sample size, it represents an ongoing project designed to expand the dataset in the coming years. In addition to the samples still buried at La Higueruela, new burials have been established both at La Higueruela and in the Garden of the MNCN. These ongoing experiments will facilitate the characterization of root-mark morphologies produced by a broader range of plant types, including other herbaceous species, trees, and shrubs. The aim is to increase the database of this research to contribute to the identification of plants that cannot fossilize or where the burial environment may have changed, and improve interpretations in different contexts.

## Conclusions

This study has shown that root marks on buried bones vary according to the type of vegetation, time of exposure and burial depth, resulting in clear differences in the morphology, distribution, and intensity of root-induced modifications. Root marks appeared after only one year of burial, demonstrating that these results are applicable even in recent forensic contexts. From an environmental and paleoecological perspective, plants inferred from root marks recorded on skeletal elements may be considered contemporaneous with the burial of the remains or shortly thereafter. Therefore, they can serve as indicators of local vegetation at the time of burial, and be used to compare with the animal’s diet, reflect climatic variations, and provide valuable environmental information. In addition, we provide a clear classification and description of root marks, facilitating their differentiation from other taphonomic modifications, such as cut marks or tooth marks. This research constitutes an ongoing experimental framework. Future studies incorporating additional plant species will increase the number of bone samples and extend exposure times, allowing further refinement of root-mark traits and their association with specific vegetation types. This will strengthen the interpretive value of root marks in paleoecological, archaeological, and forensic contexts.
